# Appendicitis1Read at the forty-second annual meeting of the Medical Association of Central New York, held at Auburn, October 19, 1909.

**Published:** 1910-03

**Authors:** Albert L. Beahan

**Affiliations:** Canandaigua, N. Y.


### Appendicitis[1]

By ALBERT L. BEAHAN, M. D., Canandaigua, N. Y.

THE general practitioner, the internist, the surgeon and, in a measure, all medical specialists must review from time to time the appendicitis proposition. The question comes with force at the patient's bedside, what is the best way to meet the conditions found in this particular case? A life is to be saved or lost on the judgment of the moment from the plan to be followed. The theories or truths believed or known must be applied in the interests of the patient.

What can medicine offer? If conclusive proof that a bad appendix is present, with sharp initial symptoms of acute pain,

1. Read at the forty-second annual meeting of the Medical Association of Central New York, held at Auburn, October 19, 1909.

vomiting, and chill, no medicine should be given. Only immediate surgery can save.

Take a parallel instance of a strangulated hernia of even three hours' duration, and the included bowel may be found so dark that a question arises whether or not to put it back into the abdominal cavity. The mucosa of the appendix gives an equally rapid form of gangrenous involvement from strangulated circulation, either in patches or in total. An infective aperture opens into the abdomen. Its size and the character of the infection become at once the paramount question. The strangulated hernia shows at first a reddened, then a blackened surface and the appendix on its interior surface, possibly in all its coats, shows in patch or in all its surfaces and structures a reddening, then a black color, indicating rapid death of the part from the pressure necrosis, so well described long ago by Morris, and which parallels the picture of a strangulated hernia.

When the red pus of an early appendicitis is confined to the appendix and the bacteria are of innocent type or moderately virulent, removal of the appendix is simple and safe. But when the streptococci or a mixed infection succeed to the invasion, the chances for success are much less. The age of the patient is a prime factor. The infant and the child cannot safely wait operation because of thin walled appendix in early life and increased danger of rapid perforation. Above forty-five years of age, as Price has determined, all pus cases should be looked upon with dread because the mortality will be high.

What symptoms tell the dangers? No one unit, nor complex, nor composite group can at a moment's notice be relied upon. If the appendix when inflamed becomes agglutinated by its own serous exudate from infection to a part, fever will follow in direct relation to the number of lymphatics found at or near the point of its attachment and thus promote infective absorption. On the shelf of the ilium there will be no fever because no lymphatics are there located; directly downward slight fever, in the other directions upward or inward more fever, with the highest fever where the region of the small intestine gets into the running, or at just above the ileocecal valve region.

If gangrene develops, pain stops and vomiting ceases for a time. A critical, labored, painstaking consideration of this epoch with a right determination means life or death to the patient. Operation is the only salvation for the life and at once. Ordinarily in the majority of cases a wait of two to four days can be made to let the case develop such symptoms as shall help in discriminating for or against operation. Sometimes the old rule of fracture in surgery, should be considered, that is, to always call another surgeon to avoid malpractice, to make sure one was right. No definite final fact can be fixed in the mind. It depends on the degree of the strangulated tissue involved which is impossible to estimate even to imagine. Oftentimes what is occurring in the appendix tissue is a conundrum. The inspection cannot be made as is the case in the strangulated hernia. Following a series of cases of operation and viewing the pathology will wonderfully quicken the understanding and correct the reckonings regarding the true findings. If the initial pain is slight and nausea comes but no vomiting and chill be absent, the probability of appendicular or colon colic is to be considered as a cause for the manifestations present. If pain, vomiting, or chill have been followed by rapid pulse and rising temperature, no hesitation of prompt surgical interference should be felt. Perhaps the abdominal symptoms alone should guide. My own experience confirms this conclusion and conviction. Persistent right or left or general abdominal rigidity, even if slight, points to nature's splinting a sore appendix and trying to so place the organ at rest that her abdominal guard, the omentum, can house from doing more harm to the poisoned appendix. The appendicitis gait, so aptly described by Dr. Wheeler of East Bloomfield, N. Y., walking with the right leg as if splinted from the pelvis, is significant of nature's intent to stop flexion, or extension of the leg on the thigh and give added rest to the inflamed appendix.

The diagnosis of appendicitis and the need of operation can be enunciated by the educated finger tips alone. Find out what nature is trying to do from the expression of the effort at abdominal fixation, and an infallible guide for or against operative procedure is at hand. Possibly the assumption of procrastinators and their teachings of certain periods of safety or non-safety, may influence the determination of minds following the trend of published works on this subject, but the simple truth seems to be in favor of open knowledge by open incision rather than late, as a rule, with very carefully selected exceptions. Refusal to operate in desperate cases may place some life away from the mortality statistics of an operator, but in the mortuary statistics of the state reports, a ruptured gall-bladder or abscess of surgical kidney or Potts spinal abscess, or pelvic pus formation, will be undiscovered while only the light of an open incision of the abdomen can develop therein a saving technic.

It needs the training of a hospital conducted carefully on procrastinating lines only where ultra bad cases are never operated. but die untouched by surgeons in this a surgical disease, to receive appendicitis cases and handle them in this way. It is not in homes that it can be done? Is it honest surgery? The stomach washing, the blood counts and analysing, the starvation and posturing are not applicable to the country or village practice, to the masses of the city, to the private hospital, where the fact is not well developed and practised to a nicety and precision of detail above and beyond the conception of the rank and file of the profession.

These teachings befog the medical mind. The waiting plan with the morphine paralysing adjunct, with stomach lavage, starvation and rest is criminal practice in many cases, as it gives inoperative conditions later and immediate dangers. The safe rule in certain appendiceal inflammation cases is immediate operable interference and to do no harm by covering with opium in any form the natural expressions of pain and spasm, these talismen of natural expressions of need of help. Greater haste, is required as in the parallel of strangulated hernia, not resting in any delusive hope of a nature cure. Repeated minor attacks of appendicitis, producing the fibrous nerve reflex pathological appendix, as described by Morris, gives a pernicious influence on the nervous system. Removal of the appendix restores the nervous system's equilibrium in a satisfactory and beautiful way.

Chronic appendicitis and the fibrous appendix induces in both sexes serious nervous disturbances. In the female, when the appendix is involved in disease in childhood, an additional reason for removal in moderately urgent cases, is the certain ovarian disease especially of the ovarian capsule. The presence of ovarian hematocele because of this thickening of the ovarian envelope is, in our experience, frequently found in the appendicitis in adolescent girls; and right tube ectopic gestation associated with an old appendicitis has been encountered too often to be a coincidence in women. In two of these cases when removal of appendix was made they quickly developed pregnancy from the left side.

For this reason a female child deserves emphatic opportunity to be rid of a diseased appendix with much less reason than a male child. The child's future as a woman is bound up in this proposition, for both opportunity for perpetuation of her kind and to escape the neurasthenia or the knife so frequently the only later possibility of a neglected condition. Several of the communities contiguous to the hospital I serve have been shorn of old appendicitis cases and new cases are quite rare. This gives comfort to the physician in these favored communities and contributes to the well being of the people so happily and skilfully served. Repeated attacks of appendicitis do not worry and disturb these physicians. They induce early operation. They clean up their field and do not permit their followers to dally and fumble. Their mortality is nil, their reputations are of the best.

This result is better than waiting and talking soft words to willing ears but blind prejudices. The reasoning and practice which brings this result to a country practice is not to be gainsaid by any individual, any hospital, any aggregation of men who teach and practise the nature relief plans.

The false notions permeating the profession of postponed or protracted periods of delay, cannot be held in mind by physicians of this experience with their own successful efforts of eradication by prompt efficient early work. What surgeon would not be a midnight operator braving weather and meeting small preparations in simple homes, if with no mortality? The children coming on the laps of physicians on Sundays and after dark who recover is a compliment to the early diagnostic acumen of their physicians, and become a laurel leaf to the brow of the diligent opportune surgical worker. It is a safe, sane and successful policy and it becomes, with very few exceptions, the working formula of a surgeon to the manor born, who becomes after many years shorn in mind of fads and foibles and learns the lesson taught by the unequivocal naked truth.

